# Comparison of correction of kyphotic deformity and implant failure in percutaneous short-segment pedicle screws fixation with index level versus long-segment pedicle screws fixation without index level for traumatic thoracolumbar junctional fractures: A prospective cohort study

**DOI:** 10.12669/pjms.40.12(PINS).11110

**Published:** 2024-12

**Authors:** Muhammad Jehanzeb, Ahtesham Khizar, Muhammad Asif Shabbir, Muhammad Shakir, Khawar Anwar, Asif Bashir

**Affiliations:** 1 Dr. Muhammad Jehanzeb, MBBS, MS Senior Registrar Neurosurgery, Department of Neurosurgery Unit-I, Punjab Institute of Neurosciences, Lahore, Pakistan; 2 Dr. Ahtesham Khizar, MBBS, FCPS Senior Registrar Neurosurgery, Department of Neurosurgery Unit-I, Punjab Institute of Neurosciences, Lahore, Pakistan; 3 Dr. Muhammad Asif Shabbir, MBBS, FCPS Associate Professor Neurosurgery, Department of Neurosurgery Unit-I, Punjab Institute of Neurosciences, Lahore, Pakistan; 4 Dr. Muhammad Shakir, MBBS, FCPS Assistant Professor Neurosurgery, Department of Neurosurgery Unit-I, Punjab Institute of Neurosciences, Lahore, Pakistan; 5 Dr. Khawar Anwar, MBBS, MS Senior Registrar Neurosurgery, Department of Neurosurgery Unit-I, Punjab Institute of Neurosciences, Lahore, Pakistan; 6 Prof. Dr. Asif Bashir, MD, FAANS, FACS Professor of Neurosurgery, Department of Neurosurgery Unit-I, Punjab Institute of Neurosciences, Lahore, Pakistan

**Keywords:** Spinal fractures, Kyphosis, Pedicle screws, Minimally invasive surgery, Comparative study

## Abstract

**Objective:**

To compare correction of kyphotic deformity (KD) and implant failure (IF) in percutaneous short-segment pedicle screws fixation (SSPF) with index level versus long-segment pedicle screws fixation (LSPF) without index level for traumatic thoracolumbar (TL) fractures.

**Methods:**

This prospective study comprised 56 patients who met the study’s inclusion criteria from the Department of Neurosurgery at the Punjab Institute of Neurosciences in Lahore, Pakistan presented between June 2022 and May 2023. We separated them into two groups: Group-A and Group-B, each with 28 patients. Group-A consisted of percutaneous SSPF with incorporated screws in the fractured vertebra, whereas Group-B consisted of percutaneous LSPF without index level involvement for traumatic TL fractures. We reviewed the patient’s preoperative, postoperative, and follow-up radiographs. The quantitative factors such as Cobb’s angle and implant stability were investigated.

**Results:**

The study comprised individuals with a mean age of 31.5 ± 10.6 SD years. Out of 56 patients, 38 (67.85%) were male and 18 (32.14%) were female. The fracture level distribution was 37 (67.07%) patients with L1 fracture, 15 (26.78%) with D12 fracture, 2 (3.57%) with D11 fracture, and 2 (3.57%) with L2 fracture. Group-A had a preoperative Cobb’s angle of 18.8° ± 5.0° SD, whereas Group-B had 19.8° ± 6.3° SD (P-value=0.23). Immediate postoperative Cobb’s angle was 6.4° ± 3.4° SD in Group-A and 7.3° ± 3.7° SD in Group-B (P-value 0.66). After three months, Group-A had a Cobb’s angle of 7.1° ± 3.6° SD, whereas Group-B had 7.8° ± 3.7° SD (P-value = 0.78). Six-month follow-up Cobb’s angle was 7.9° ± 3.6° SD in Group-A and 8.4° ± 3.8° SD in Group-B (P-value=0.502). There were no implant failures in any group.

**Conclusions:**

For a single level traumatic TL fracture, SSPF with index level can preserve Cobb’s angle better than LSPF without index level, and it has high IF stability.

**List of Abbreviations:**
SSPF: Short-Segment Pedicle Screws Fixation,
LSPF: Long-Segment Pedicle Screws Fixation,
TL: Thoracolumbar,
KD: Kyphotic Deformity,
IF: Implant Failure,
SD: Standard Deviation,
SPSS: Statistical Package for Social Sciences,
CT: Computed Tomography,
MRI: Magnetic Resonance Imaging.

## INTRODUCTION

Among injuries to the spine, traumatic thoracolumbar (TL) fractures are most frequent.^1^,^2^ More than 90% of spinal injury instances have traumatic etiologies, including incidents like violent crime, sports, falls, or auto accidents.^3^ Younger people regularly suffer from TL burst fractures, which are highly prevalent that may significantly affect patient’s daily physical activity and life. In addition to neurological impairment, these fractures are typically accompanied by kyphotic deformity (KD). TL fracture treatment aims to restore mechanical stability to the fracture and trigger neurologic healing, allowing patients to return to work. The management and treatment options of unstable TL spine fractures are debatable despite their prevalence.^1^,^2^ TL fracture treatment has made significant strides. In clinical practice, posterior pedicle screw fixation is frequently employed and offers biomechanically three column fixations. Since then, a variety of surgical procedures are developed for the management and treatment of TL spine burst fractures which includes short-segment pedicle screws fixation (SSPF) or long segment pedicle screws fixation (LSPF), corpectomy and direct anterior decompression of spine and combined approach which is anterior and posterior.^4^

The techniques of treatment, however, are still up for debate.^5^ There is disagreement about whether using short or long posterior fixation and which is the best course of action for treating TL burst fractures.^6^-^8^ LSPF (two levels above and below the fracture level) offers better implant stability; nevertheless, this causes potential superfluous instrumentation and increases the pressure on the lower discs.^9^ A short posterior fixation, contrasted with, limits the number of fused vertebral segments and utilizes pedicle screws and connected rods one level above and below the fractured vertebra to prevent excessive loads on the adjacent discs. There have been reports on using posterior fixation for burst fractures, but there are substantial rates of fixation failure and kyphotic collapse in posterior spine fixation.^10^-^12^ The standard treatment for fracture-dislocations has been long segment posterior fixation. But in both techniques Post operative neurology improved and deformity was corrected.

Due to superior implant stability, longer segment fixation (two levels above and below) is better suitable for a posterior-only strategy in the therapy of these fractures.^13^ The correction in kyphosis and maintenance of spine sagittal alignment are achieved by incorporating the fractured vertebra in a SSPF for the fractures of TL junction, much as LSPF. The two groups’ neurological outcomes were comparable as well. Eventually, this method enabled the preservation of two or more spinal motion segments.^14^ Several studies have previously demonstrated the benefits of employing index screws in fractured vertebrae for short-segment posterior fixation in burst fracture instances to improve correction and preserve fusion segments.^7^,^12^ There are not many studies, nevertheless, on TL spine fracture dislocation and short-segment posterior trans pedicle fixation.^15^,^16^ The aim of this study was to analyses and compare percutaneous SSPF with integrated screws at the level of fractured vertebra with percutaneous LSPF without index level for the correction of kyphosis and implant failure (IF) in TL spine burst fractures.

## METHODS

This prospective cohort study was conducted at the Department of Neurosurgery, Punjab Institute of Neurosciences, Lahore, Pakistan. The study duration was 12 months, 1^st^ June 2022 to 31^st^ May 2023

We did a non-probability based consecutive sampling. In our sample total of 56 patients were included with each group including 28 patients. The sample size for the study was calculated by the following formula keeping the power of study equal to 80% and confidence level equal to 95% with expected mean KD correction in Group-A (SSPF) as 13.2° ± 5.2° SD and 16.6° ± 4.2° SD in Group-B (LSPF). 17

n = (Z_1-β_+Z_1-a/2_)^2^(ó_1_^2^+ó_2_^2^)/(ų_1_-ų_2_)^2^

**Z_1-β_ = ** Desired power of study (80%) = 0.84 

**Z_1-a/2_ = ** Desired confidence level (95%) = 1.96

**ų1 = ** Anticipated mean KD correction in Group-A = 13.2

**ų2 = ** Anticipated mean KD correction in Group-B = 16.6

**ó1 = ** Standard deviation of KD correction in Group-A = 5.2

**ó2 = ** Standard deviation of KD correction in Group-B = 4.2

**n = ** Calculated sample size in each group = 28

### Ethical Approval:

This study was approved by IRB with reference no. 00/168/22/PGMI/LGH.

### Inclusion Criteria:

• Patients with TLICS ≥ 4
• Single Level Fractures involving thoracolumbar fractures from T11 – L3
• Patients with thoracolumbar vertebrae fracture with intact pedicle on CT scan
• Both genders
• Age 20 to 60
• AO spine Fracture Type A (A2, A3, A4) and (B1, B2, B3)

### Exclusion Criteria:

• Multilevel spinal injury
• Osteoporotic fracture
• Pathological fracture
• Patient with vertebral fracture pedicle on CT
• AO spine Fracture Type C
• In which decompression was needed secondary to retropulsion

### Surgical procedure:

### Short Segment Pedicle Screw Fixation (SSPF):

**Fig.1:**
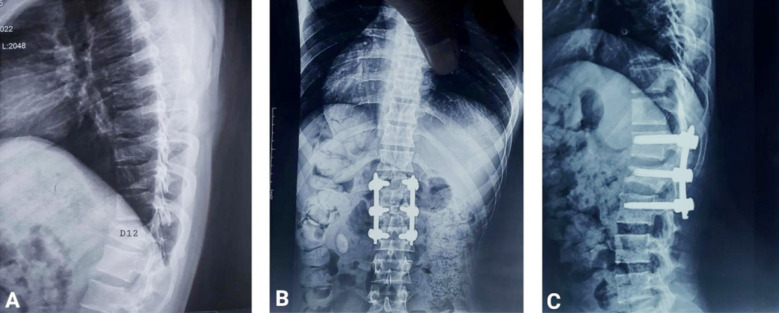
A: Preoperative X-ray dorsal spine lateral view showing D12 fracture, B&C: Postoperative X-rays AP view and lateral views for SSPF.

Under General Anesthesia (GA), the patient was positioned face down, full aseptic measures were taken, and C-ARM was used to identify the pedicles of the appropriate vertebrae. Over the pedicles, multiple 1-1.5 cm skin incisions were made, and the TL fascia was incised. With the use of the C-ARM, Jamshidi needles were placed into the pedicles, and a guide wire was then threaded through it. The Jamshidi needles were taken out after the guide wire was inserted, then dilators were passed over the guide wire. Later, utilizing cannulated instruments, such as taps, the pedicle preparation process was initiated. The depth of the instrument was evaluated on lateral imaging. The guide wire was withdrawn after the pedicle screws were introduced in the vertebral body, as seen on the lateral radiograph, and screws were then inserted one level above, one level below, and in the fracture vertebra (index level). Pre-bend rod was inserted into screws after all of the pedicle screws were in place. Under the C-arm, the screw was deflected, and the rod was fixed. For the purpose of verifying the construct, lateral and anterior-posterior final imaging were collected as shown in Fig.1. Skin and fascia were approximated. It was covered with an antiseptic dressing.

### Long Segment Pedicle Screw Fixation (LSPF):

**Fig.2:**
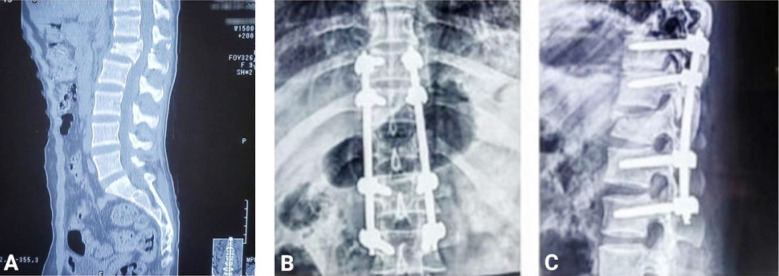
A: Preoperative CT TL spine showing L1 fracture, B&C: Postoperative X-rays AP and lateral views for LSPF.

Under GA, the patient was positioned face down, full aseptic measures were taken, and C-ARM was used to identify the pedicles of the appropriate vertebrae. Over the pedicles, multiple 1-1.5 cm skin incisions were made, and the TL fascia was incised. With the use of the C-ARM, Jamshidi needles were placed into the pedicles, and a guide wire was then threaded through it. The Jamshidi needles were taken out after the guide wire was inserted, and dilators were then passed over the guide wire. Later, utilizing cannulated instruments, such as taps, the pedicle preparation process was initiated. The depth of the instrument was evaluated on lateral imaging. The guide wire was withdrawn after the pedicle screws were introduced in the vertebral body, as seen on the lateral radiograph, and screws were then inserted two levels above, two levels below. Pre-bend rod was inserted into screws after all of the pedicle screws were in place. Under the C-arm, the screw was deflected, and the rod was fixed. For the purpose of verifying the construct, lateral and anterior-posterior final imaging were collected as shown in Fig.2. Skin and fascia were approximated. It was covered with an antiseptic dressing.

### Data Collection Procedure:

Following approval from the institution’s ethical committee, 56 patients from the Department of Neurosurgery Unit-I at Punjab Institute of Neurosciences, Lahore, Pakistan were included in the study who met the inclusion criteria and underwent TL fracture treatment with either SSPF or LSPF. Age, gender and demographic information were documented. Patients received a thorough explanation of the pros and cons of participating in the study, and informed consent was acquired. Both the groups underwent non probability based consecutive sampling. 28 patients underwent percutaneous SSPF screws (Group-A) with index level (6 screws) while 28 patients underwent percutaneous LSPF (Group-B) without index level involvement (8 screws). The study comprised patients with single-level burst fracture of the TL spine. To determine neurological deficiency according to the American Spinal Injury Association (ASIA) scale, a clinical and neurological examination was conducted. X-rays, baseline computed tomography (CT) of the injured area, and magnetic resonance imaging (MRI) for fracture assessment were performed in all patients. We used the Cobb’s angle method to measure the kyphotic angle. Patients were mobilised on the second postoperative day. The postoperative issues were noted. Following up with patients allowed for the recording of implant stability, neurological, functional, and radiological outcomes. At the follow-up visit, each patient’s neurological condition was assessed using the ASIA score. By measuring the Cobb’s angle and using postoperative and final X-ray for follow-up, the surgical result was assessed. During postoperative and final follow-up, the loss of KD correction and implant stability were also noted. All patients underwent postoperative radiological evolution. The correction of Cobb’s angle and IF were assessed on the postoperative radiographs, and follow-up radiographs. Results from radiographic analysis performed before and after surgical procedure were analysed and compared. Follow-up of the patients was done at three months and six months after discharge.

Implant failure is defined as the fracture or unintended displacement of a metal component, such as a screw or rod, or the disassembly of a fixed construct. This includes screw pull-out, which is the translation of a pedicle screw parallel to its long axis, and screw/rod breakage, where the fracture occurs anywhere along the length of the screw or rod. Additionally, screw loosening is identified by a radiologically discernible radiolucent halo around the screw, with a halo of more than 1 mm considered a definitive sign of loosening. These criteria are typically assessed within a 6-month follow-up period.

### Statistical Analysis:

Statistical Package for Social Sciences (SPSS) version 25.0 was used to enter and evaluate the data. Age, Cobb’s angle, and implant stability were among the quantitative characteristics that were provided as mean standard deviation (SD). The frequency and percentage values for the qualitative factors, such as gender and implant failure, were provided. Using the Student-test, the differences in age, gender, fracture severity, and Cobb’s angle correction between the two groups were compared. The chi-square test was used to compare IF rates between the two groups. Statistical significance was determined by a P-value of less than 0.05.

## RESULTS

In this study, 28 patients in Group-A underwent SSPF incorporating fractured vertebrae. There were 18 (64.28%) patients in the age range of 20 to 30 years, 5 (17.85%) in the range of 31 to 40 years, 3 (10.71%) in the range of 41 to 50 years, and 2 (7.14%) in the range of 51 to 60 years. 28 patients were included in Group-B, who underwent LSPF, 18 (64.28%) with age range of 20 to 30 years, 7 (25%) were in the range of 31 to 40 years, 2 (7.14%) were in the range of 41 to 50 years, and 1 (3.57%) was in the age range of 51 to 60 years. Mean age of patients in Group-A and Group-B was 32.3 ± 11.8 SD years and 30.7 ± 9.4 SD years, respectively. P-value was 0.241 (Table-I).

**Table-I:** Age distribution of patients among both the groups.

Age (years)	Group-A (SSPF) n=28		Group-B (LSPF) n=28		P-value

	No. of patients	Percentage (%)	No. of patients	Percentage (%)	
20-30	18	64.28	18	64.28	0.241
31-40	5	17.85	7	25	
41-50	3	10.71	2	7.14	
51-60	2	7.14	1	3.57	
Mean ± SD	32.3 ± 11.8 years		30.7 ± 9.4 years		

### Gender Distribution:

Out of 56 patients, 38 patients (67.85%) were male and 18 patients (32.14%) were female (Table-II). In Group-A, 17 patients (60.71%) were male and 11 patients (39.28%) were female. Similarly, in Group-B, 21 patients (75%) were male and seven patients (25%) were female, (Table-II).

**Table-II:** Gender distribution of patients among both the groups.

Gender	Group-A	Group-B	Total
Male	17 (60.71%)	21 (75%)	38 (67.85%)
Female	11 (39.29%)	7 (25%)	18 (32.15%)
Total	28 (100%)	28 (100%)	56 (100%)

### Vertebral Fracture Level Distribution:

In this study, we included TL fractures from D11 to L2 and there were 37 patients (67.07%) with L1 fracture, 15 patients (26.785 %) with D12 fracture, 2 patients (3.57%) with D11 fracture and 2 patients (3.57%) with L2 fracture, (Table-III).

**Table-III:** Vertebral fracture level distribution among patients of both the groups.

Fracture Level	Group-A	Group-B	Total	Percentage
D11	2	0	2	3.57%
D12	7	8	15	26.78%
L1	17	20	37	67.07%
L2	2	0	2	3.57%

### Neurological outcome:

We analyzed preoperative and postoperative neurology in accordance with ASIA scores. Two patients in Group-A (SSPF) and three patients in Group-B (LSPF) improved after surgery. There was no neurologic deterioration in both the groups. (Table-IV)

**Table-IV:** Comparison of neurological status by ASIA score among both the groups.

ASIA	Group-A		Group-B	

	Preoperative	Postoperative	Preoperative	Postoperative
A	0	0	0	0
B	2	2	1	1
C	4	4	2	2
D	7	5	6	3
E	15	17	19	22

### Outcome of kyphotic deformity correction and implant stability:

**Fig3:**
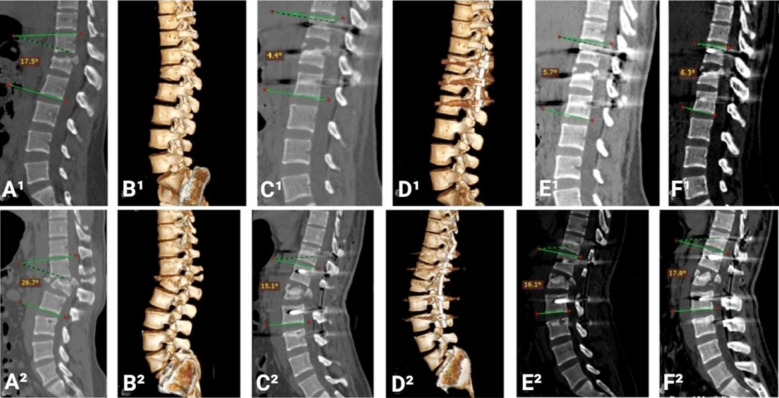
Cobb’s angle measurements, A^1^,B^1^: Preoperative, C^1^,D^1^: Immediate postoperative, E^1^: 3-month follow-up, F^1^: 6-month follow-up for SSPF. A^2^,B^2^: Preoperative, C^2^,D^2^: Immediate postoperative, E^2^: 3-month follow-up, F^2^: 6-month follow-up for LSPF.

Preoperative, postoperative and follow-up Cobb’s angles were measured and compared in this study as shown in Fig.3. Preoperative Cobb’s angle noted in Group-A was 18.8° ± 5.0° and in Group-B 19.8° ± 6.3° with P-value 0.231, which was not significant statistically. Immediate postoperative Cobb’s angle was 6.4° ± 3.4° in Group-A and 7.3° ± 3.7° in Group-B with P-value 0.662, which was not significant statistically. Three months follow-up Cobb’s angle was 7.1° ± 3.6° in Group-A and 7.8° ± 3.7° in Group-B with P-value 0.783, which was not significant statistically. Six months follow-up Cobb’s angle was 7.9° ± 3.6°in Group-A and 8.4° ± 3.8° in Group-B with P-value 0.504, which was not statistically significant. (Table-V) (Figure. 3). There was not a single implant failure in any of group in 12 months follow-up.

**Table-V:** Outcome of kyphotic deformity correction at preoperative, postoperative, 3-month follow-up and 6-month follow-up period.

Cobb’s angle	Group-A	Group-B	P-value
Preoperative	18.8° ± 5.0°	19.8° ± 6.3°	0.231
Postoperative	6.4° ± 3.4°	7.3° ± 3.7°	0.662
3-month follow-up	7.1° ± 3.6°	7.8° ± 3.7°	0.783
6-month follow-up	7.9° ± 3.6°	8.4° ± 3.8°	0.504

## DISCUSSION

The study’s findings demonstrate that both the groups experienced fast surgical correction, which was reduced at the 3-month follow-up but was maintained with some angle drop at the 6-month follow-up. Hence, the correction of the Cobb’s angle was equally effective with both approaches.

TL fractures, especially burst fractures are more common in younger age groups and significantly affect their daily physical activity.^18^ They commonly result in neurological deficits and KD. Regardless of the approach used, the goals of therapy for a TL burst fracture are early mobilisation and rehabilitation by restoring the mechanical stability of the fracture and inducing neurologic recovery, allowing patients to go back to work. In the current study, percutaneous SSPF with index level involvement was compared with LSPF without index level involvement in terms of KD correction and IF in TL burst fractures management. The average age of the patients included in this study was 31.5 ± 10.6 SD years, and the average age of patients in Group-A (SSPF) and Group-B (LSPF) was 32.3 ± 11.8 SD years and 30.7 ± 9.4 SD years, respectively. A similar kind of study was conducted recently which included 26 patients with mean age 29.8 years and age range from 19-50 years.^19^ In our study, 38 patients (67.85%) were male and 18 patients (32.14%) were female. Male patients 17 (60.71%) and 11 patients (39.28%) were female in Group-A whereas in Group-B, 21 patients (75%) were male and seven patients (25%) were female. Similarly, the above mentioned study included 26 patients in which there were 20 (76.92 %) male and 6 (23.07%) female.^19^ The study’s findings support other authors’ findings that the male population is at high risk. This might be because men are more likely to have road traffic accidents and falls from height. The average age of the patients shows that TL vertebral trauma is more common in patients under the age of 50. This may be because road traffic accidents frequently result in trauma in Pakistan.

Mean Cobb’s angle preoperatively in Group-A was 18.8° ± 5.0° and in Group-B was 19.8° ± 6.3° which improved to 6.4° ± 3.4° in Group-A and 7.3° ± 3.7° in Group-B. After six months follow-up Cobb’s angle was maintained but a drop to 7.9° ± 3.6° in Group-A and 8.4° ± 3.8° in Group-B was noted with P-value 0.521. A study by El Behairy HF et al.^20^ demonstrated that the Cobb’s angle before surgery was 25° ± 8.0°, which reduced to 9.0°± 7.0° in the immediate postoperative period. There was a mean loss of 2.5° noted at the final follow-up examination. This study favors the results of our study. Mittal et al.^19^ described in his study about the outcome of Cobb’s angle preoperatively and postoperatively. Their study consisted of 50 patients in which preoperative Cobb’s angle was 26.17° ± 7.338°, 16.29° ± 7.568°, immediate postoperative Cobb’s angle was 7.75° ± 5.738°, 6.93° ± 6.22°, and final follow-up Cobb’s angle was 11.58° ± 5.198°, 9.23° ± 7.18° in Groups-1 (LSF) and 2 (SSF), respectively. These results are comparable with our study.

In 2020 Singh et al.^17^ in his study showed that in the postoperative period, the KD correction was comparable between the two groups. Loss of kyphosis correction between Group-A is equivalent at the one year follow-up to Group-B, while in Group-A preoperative Cobb’s angle was 13.2° ± 5.2° and in Group-B was 16.8° ± 4.2°, postoperative Cobb’s angle in A group was 7.1° ± 7.4° and in Group-B was 6.2° ±6.3°, in their results. When compared to LSPF, SSPF is associated with much less surgical time and blood loss. Both the groups’ functional and neurological outcomes are comparable. An index screw or anterior column reconstruction may help SSPF patients to retain more of their corrected kyphosis.

In another study, the average preoperative Cobb’s angle was 20.96° ± 4.74° in Group-1, while in Group-2 it was 22.59° ± 5.89° (P=0.234). Postoperative correction was somewhat improved in Group-1 than in Group-2. In fact, the mean postoperative Cobb’s angle was 14.2° ± 6.50° for Group-1 and 17.13° ± 11.63° for Group-2, with mean correction of 6.73° and 5.46°, respectively. Although not statistically significant (P=0.243), this difference was nonetheless present. Also, at the most recent follow-up, there was no discernible difference in the corrective loss between the two groups. In actuality, the mean Cobb’s angle at the most recent follow-up was 15.97° ± 5.62° for Group-1 and 17.76° ± 11.22° for Group-2. Corrective loss in Group-2 was 0.63° lesser than in Group-1 (1.74°). However, this difference lacked statistical significance (P=0.427).^14^

While assessing the stability of the index level fixation, several biomechanical investigations have been conducted. These studies verified that short segment fixation with intermediate screw constructs are superior to traditional short segment constructs. It had been demonstrated that using level screws with fractures reduced the loads on the construct’s superior and inferior pedicle screws.^21^ Authors of various studies on burst fractures have supported the use of index screws in fractured vertebrae, which provide good stability.^7^,^14^,^22^ Fifty patients with TL fracture-dislocation were treated by Chokshi et al.^15^ in 2019 using a short-segment construct with index screws. They came to the conclusion that satisfactory KD correction and maintenance can be achieved with short-segment fixation for TL fracture-dislocation. No group in our study experienced an IF throughout the six-month follow-up period which is comparable to other studies. In his work, “outcomes of TL fracture-dislocation handled by short-segment and long-segment posterior fixation”, Mittal et al.^19^ (2021) included 52 patients, and at follow-up, neither group had even one instance of pseudoarthrosis, IF, or screw breakage. The radiological results showed no statistically significant difference between the two groups.

In 2016 Dobran et al.^14^ examined 60 patients in the treatment of unstable TL junction fractures and found no implant failures, including screw breakages or loosening, in either group. They compared long-segment instrumentation with short-segment pedicle fixation with inclusion of the fracture level. Therefore, this favors the management of TL burst fractures with percutaneous SSPF with incorporated screws at the level of fractured vertebra and LSPF in terms of IF with good stability and very low IF probability. This treatment of TL burst fractures is being compared for the first time in our country in this current study with percutaneous SSPF with integrated pedicle screws at the level of fracture compared to LSPF in terms of KD correction and IF.

### Limitations:

The limitation are small sample size, single center design and short follow-up period. These limitations suggest caution in generalizing the findings and highlight the need for further research with larger, more diverse samples and longer follow-up periods.

## CONCLUSIONS

For the treatment of traumatic TL fractures, percutaneous SSPF with incorporated screws at the level of fracture has hardly any noticeable difference from LSPF in terms of KD correction and IF. For a single level fracture, SSPF with index level involvement can maintain the Cobb’s angle just as well as LSPF with good stability in terms of IF. None of the techniques was found superior. However, in comparison to LSPF, SSPF with integrated screws at the level of fracture vertebra preserves more mobility segments and has decreased costs of two less percutaneous screws.

### Authors’ Contribution:

**MJ:** Conception and design of study, data acquisition, manuscript writing and literature review.

**AK:** Data acquisition, manuscript writing and literature review.

**MAS, MS and KA:** Data analysis, Interpretation of data, and Manuscript editing.

**AB:** Supervision and critical review.

All the authors have read and approved the final manuscript and are responsible and accountable for the accuracy and integrity of the work.
